# Characterisation of neonatal seizures and their treatment using continuous EEG monitoring: a multicentre experience

**DOI:** 10.1136/archdischild-2018-315624

**Published:** 2018-11-24

**Authors:** Janet M Rennie, Linda S de Vries, Mats Blennow, Adrienne Foran, Divyen K Shah, Vicki Livingstone, Alexander C van Huffelen, Sean R Mathieson, Elena Pavlidis, Lauren C Weeke, Mona C Toet, Mikael Finder, Raga Mallika Pinnamaneni, Deirdre M Murray, Anthony C Ryan, William P Marnane, Geraldine B Boylan

**Affiliations:** 1 Institute of Women’s Health University College London, London, UK; 2 University Medical Center Utrecht, Utrecht, The Netherlands; 3 Department of Neonatology, Karolinska University Hospital, Stockholm, Sweden; 4 CLINTEC, Karolinska Institute, Solna, Sweden; 5 Rotunda Hospital, Dublin, Ireland; 6 Royal London Hospital, London, UK; 7 Queen Mary University of London, London, UK; 8 Irish Centre for Fetal and Neonatal Translational Research (INFANT), Cork, Ireland; 9 Department of Paediatrics and Child Health, University College Cork, Cork, Ireland; 10 Clinical Neurophysiology, University Medical Center Utrecht, Utrecht, The Netherlands

**Keywords:** clin neurophysiology, neonatology, seizures, antiepileptic drug, EEG

## Abstract

**Objective:**

The aim of this multicentre study was to describe detailed characteristics of electrographic seizures in a cohort of neonates monitored with multichannel continuous electroencephalography (cEEG) in 6 European centres.

**Methods:**

Neonates of at least 36 weeks of gestation who required cEEG monitoring for clinical concerns were eligible, and were enrolled prospectively over 2 years from June 2013. Additional retrospective data were available from two centres for January 2011 to February 2014. Clinical data and EEGs were reviewed by expert neurophysiologists through a central server.

**Results:**

Of 214 neonates who had recordings suitable for analysis, EEG seizures were confirmed in 75 (35%). The most common cause was hypoxic-ischaemic encephalopathy (44/75, 59%), followed by metabolic/genetic disorders (16/75, 21%) and stroke (10/75, 13%). The median number of seizures was 24 (IQR 9–51), and the median maximum hourly seizure burden in minutes per hour (MSB) was 21 min (IQR 11–32), with 21 (28%) having status epilepticus defined as MSB>30 min/hour. MSB developed later in neonates with a metabolic/genetic disorder. Over half (112/214, 52%) of the neonates were given at least one antiepileptic drug (AED) and both overtreatment and undertreatment was evident. When EEG monitoring was ongoing, 27 neonates (19%) with no electrographic seizures received AEDs. Fourteen neonates (19%) who did have electrographic seizures during cEEG monitoring did not receive an AED.

**Conclusions:**

Our results show that even with access to cEEG monitoring, neonatal seizures are frequent, difficult to recognise and difficult to treat.

**Oberservation study number:**

NCT02160171

**What is already known on this topic?**

- Neonatal seizures are difficult to detect clinically, and interpretation of the neonatal electroencephalography (EEG) is a highly specialised skill.
- The seizure burden is often high, particularly in neonates with hypoxic-ischaemic encephalopathy or stroke.

**What this study adds?**

- This is the first European-wide examination of continuous EEG monitoring of neonatal seizures in a large cohort.
- This multicentre EEG study showed that critically ill neonates sustain a high burden of seizures.
- Both underdiagnosis and overdiagnosis of seizures was common, despite availability of continuous EEG monitoring.
- When antiepileptic drugs were given, only 11% were given within 1 hour of the electrographic seizure episode occurring.

## Introduction

Substantial technological advances have facilitated prolonged continuous electroencephalography (cEEG) recording, but the diagnosis and management of neonatal seizures remains challenging. Interpretation of the neonatal EEG is a highly specialised skill. Very few neonatal units have rapid access to expert neurophysiology opinion, although the majority have equipment suitable for amplitude integrated EEG (aEEG) or cEEG monitoring.^1^ In future, automated seizure detection should inform clinical decision making and reduce the time required for experts to review long recordings.^2^ Prolonged cEEG recordings have confirmed the extent of the mismatch between clinical seizures and the electrographic seizure burden,^3^ and have revealed the true extent of the seizure burden present in many neurologically sick neonates. cEEG is currently recommended by the American Clinical Neurophysiology Society for neonates.^4^ A recent multicentre cEEG study in the USA found that 59% of neonates with clinically suspected seizures who were monitored for at least 24 hours had more than seven seizures, and 16% developed status epilepticus (SE).^5^

The subtle nature of clinical seizures in neonates leads to both overdiagnosis and under-recognition. The problem of ‘clinically silent’ or ‘electrographic only’ seizures (electroclinical dissociation) is particularly prevalent in this age group. The mismatch increases after the administration of antiepileptic drugs, when it has been termed ‘uncoupling’.^6^ Current estimates suggest that the mortality among neonates with seizures is around 10%, and 50% of those who survive have a significant disability.^7 8^ The debate regarding whether or not to treat to electrographic seizure quiescence, and whether achieving this would improve the prognosis, continues.^9 10^ Treatment on the basis of clinical diagnosis alone is the current standard of care in many neonatal units, but this policy carries risks. Undertreatment may lead to ‘kindling’ of additional seizures, adding to any pre-existing brain injury, and permanently altering seizure thresholds in the brain.^11^ On the other hand, overtreatment of neonates with antiepileptic drugs carries the risk of using neurotoxic medications, prolonging intensive care (with associated costs and parental separation) and exposing the newborn to potential complications from intensive care procedures or sedation.

The aim of this multicentre study was to describe detailed characteristics of electrographic seizures in a large cohort of term neonates in Europe monitored with cEEG. A secondary aim was to establish how and when seizures were treated during cEEG monitoring. We also aimed to establish a European network with shared definitions and a common platform for EEG and data collection, in order to facilitate future collaborative multicentre research in this important area.

## Methods

### Study design and participants

This was a multicentre study in six neonatal intensive care units (NICUs) across four European countries (Ireland, The Netherlands, Sweden, the UK). All neonates of at least 36 weeks gestation who required EEG monitoring for clinical purposes (including neonatal encephalopathy, suspected stroke, intracranial infection and unusual movement patterns) were eligible for inclusion in the study. Retrospective data were available from two sites for neonates enrolled between January 2011 and February 2014. Prospective data were collected from all six sites with enrolment between June 2013 and June 2015. Neonates with <6 hours of good-quality EEG recording were excluded. Written informed consent was obtained from at least one parent/guardian.

At each centre, clinical data for each neonate were entered online using a secure database system designed specifically for this study (MedSciNet, Stockholm, Sweden) by the local investigator, research fellow or research nurse. Clinical data collected included delivery details, information on the neonatal course and final primary diagnosis. The timing and types of antiepileptic drugs (AEDs) administered, if any, were also recorded.

### EEG recording and analysis

All neonates were monitored with cEEG (NicoletOne ICU Monitor & Xltek EEG, Natus, USA or Nihon Kohden Neurofax EEG-1200, Japan). cEEG monitoring commenced as soon as possible after birth and was continued for up to 72 hours where possible. Multichannel EEG recordings were obtained with active electrodes located at F3, F4, C3, C4, T3, T4, O1, O2 (or P3 and P4) and Cz, according to the international 10–20 system adjusted for neonates, with single channel electrocardiography and respiration monitoring if possible. The aEEG signal was also displayed simultaneously, usually with two channels. All recognised seizures in the cohort were treated when diagnosed, as per routine practice, whether this was on the basis of a clinical diagnosis or a diagnosis with aEEG or cEEG. We did not have a standard protocol for review of cEEG at any site during this study. Neonatologists primarily used the aEEG display to aid seizure treatment decisions when necessary. Immediate cEEG interpretation was not always available, but, in the case of suspected seizures on aEEG or clinical seizures, the cEEG was interpreted by local neurophysiologists when available.

Local protocols for AED administration were used at each site; however, the timing of AED administration in relation to seizure onset is never stipulated. This is largely due to the fact that detailed EEG seizure information has never been available before.

### Seizure analysis and quantification

Clinical seizures were recorded in the medical and nursing notes and seizure charts according to routine local practice. At the end of the EEG recording, EEGs were uploaded through an electronic case report form to a central EEG review server. cEEG recordings were reviewed in their entirety and all seizures were annotated by one member of a group 4 board-certified electroencephalographers, with specific expertise in neonatal EEG (GBB, SM, KVH, EP). For any difficult cases where some uncertainty existed (very few), a consensus was reached between two reviewers. All reviewers used a standard protocol for seizure annotation. An electrographic seizure was defined as a sudden repetitive, stereotyped discharge of minimum 10 s duration on one or more EEG channels with evolving frequency, amplitude and morphology.^12^ Following this analysis, summary measures of seizures over different time scales were calculated. The seizure period was defined as the time from the start of the first recorded seizure to the end of the last recorded seizure. Within this period, the total duration of all seizures (total seizure burden (TSB)) was calculated. The number of seizures and median seizure duration were also calculated. The maximum hourly seizure burden (maximum seizure burden (MSB)) in minutes per hour and the time of MSB (hours after birth) was calculated using a 1 hour window, shifted across the EEG monitoring period with a 1 min interval. SE was defined as MSB≥30 min/hour.^13^

### Seizure treatment analysis

The type, dose and timing of every AED administered to each neonate was recorded. For the purposes of assessing seizure treatment, seizures recorded on the EEG with at least 2 hours between them were considered to be separate seizure episodes. Therefore, in each EEG we created segments of the EEG called seizure episodes. These seizure episodes were of varying duration as the only criteria for a seizure episode was that it contained ongoing seizure activity with no interval longer than 2 hours.

Administration of an AED within 60 min of the start of the seizure episode was considered appropriate for the purposes of treatment analysis in this study. We would anticipate that a seizure episode should be treated within 1 hour of onset if the seizures were recognised. There are no national or international guidelines outlining how soon seizures should be treated. This is largely because until recently it was very hard to accurately identify electrographic seizures and we know that clinical recognition is inaccurate.

### Statistical analysis

Continuous variables were described using median and IQR and categorical variables using frequency and percentage. A one-sample binomial test was used to compare the proportions of male and female neonates in our study. Differences in seizure characteristics between the final diagnosis groups were investigated using the Kruskal-Wallis test. When a statistically significant difference was found, pairwise comparisons were performed using Dunn’s procedure with a Bonferroni correction for multiple comparisons. A Χ^2^ test was used to compare proportions between groups. Statistical analysis was performed using IBM SPSS Statistics (V.22.0; IBM, Armonk, New York, USA). All tests were two-sided and a p value <0.05 was considered to be statistically significant.

## Results

### Study sample

**Figure 1:**
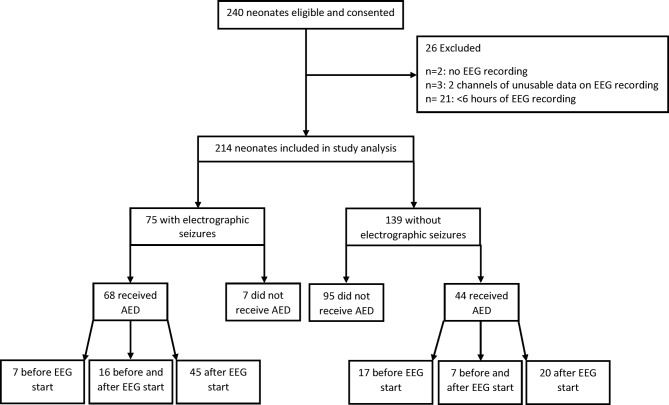
Flow diagram of recruitment, seizures and antiepileptic drug (AED) use in the cohort. EEG, electroencephalography.

Across the six sites, a total of 240 neonates were eligible and consented to participate in the study (53 from the retrospective cohort and 187 from the prospective cohort, figure 1). During the study period, all eligible infants were approached where appropriate.

Twenty-six infants were excluded from the 240 neonates eligible for this study: 2 neonates had no EEG recording available (data lost); 21 neonates had <6 hours of recording and 3 neonates had poor quality recordings. No neonates were withdrawn or died during the study period. Hence, 214 neonates were included in the study analysis (50 from the retrospective study and 164 from the prospective study) . The retrospective and prospective cohorts did not differ in terms of gestational age, birth weight or sex. The percentage of neonates with hypoxic-ischaemic encephalopathy (HIE) (any grade) was slightly higher in the retrospective cohort compared with the prospective cohort, but the difference was not statistically significant (76% vs 63%, p=0.085).

The most common reason for clinician requested EEG monitoring was HIE. Over 60% (133/214, 62%) of neonates had a primary diagnosis of HIE with 50 graded as mild, 59 as moderate and 24 as severe (table 1). A metabolic/genetic disorder was the second most common diagnosis (n=20, 9%), followed by stroke (n=18, 8%), clinically suspected seizures (n=12, 6%) and perinatal asphyxia without clinical encephalopathy (n=7, 3%). Almost three-quarters (97/133, 73%) of neonates with HIE as their final diagnosis were treated with therapeutic hypothermia (21/50 mild HIE; 52/59 moderate HIE; 24/24 severe HIE).

**Table 1:** Characteristics of neonates, n=214*

	Median (IQR)*
Gestational age at delivery (weeks)	40 (39–41)
Birth weight (g)	3435 (3110–3803)
Sex: n (%)	
Male	137 (64.0)
Female	77 (36.0)
Apgar score at 5 min (n=209)	5 (3–8)
Requirement for EEG monitoring: n (%)	
HIE	107 (50.0)
Clinician request	67 (31.3)
Stroke	7 (3.3)
Infectious	4 (1.9)
Metabolic	3 (1.4)
Other	26 (12.1)
Baby developed HIE: n (%)	
Yes	141 (65.9)
No	73 (34.1)
Clinical Sarnat score at 24 hours (n=132): n (%)	
Mild	51 (38.6)
Moderate	57 (43.2)
Severe	24 (18.2)
Therapeutic hypothermia: n (%)	
Cooled	106 (49.5)
Uncooled	108 (50.5)
Final diagnosis: n (%)	
HIE grade	
Mild	50 (23.4)
Moderate†	59 (27.6)
Severe	24 (11.2)
Metabolic/genetic disorder‡	20 (9.3)
Stroke§	18 (8.4)
Suspected seizures—unconfirmed	12 (5.6)
Perinatal asphyxia without clinical encephalopathy	7 (3.3)
Sepsis/meningitis	6 (2.8)
Intracranial haemorrhage	5 (2.3)
Other¶	13 (6.1)

*Unless otherwise stated.

†n=1 had meningitis also.

‡n=1 had severe HIE also.

§n=2 had mild HIE and n=3 had moderate HIE also.

¶n=3 for postnatal cardiorespiratory arrest (n=1 with mild HIE also, n=1 with severe HIE also); n=2  for   each of: neonatal drug withdrawal syndrome; respiratory distress; seizures of unknown origin; n=1 for each of: congenital anaemia; congenital  brain malformation; meconium aspiration syndrome; tracheo-oesphageal atresia and cystic periventricular leukomalacia  periventricular leukomalacia.

EEG, electroencephalography; HIE, hypoxic-ischaemic encephalopathy.

The characteristics of the 214 neonates included in the analysis are described in table 1. A higher percentage of neonates were male (64% male vs 36% female, p<0.001).

### EEG monitoring and seizure characteristics

EEG monitoring commenced at a median age of 8.2 hours of age (IQR 4.0–29.7) and continued for a median of 70.3 hours (IQR 31.0–95.7). Monitoring continued for at least 24 hours for the majority of neonates (173/214, 81%). Over one-third (75/214, 35%) of neonates had at least one electrographic seizure identified by the expert group. Once monitored, males and females were equally likely to have seizures identified; (34% (47/137) for males vs 36% (28/77) for females, p=0.762). The principle diagnosis of the neonates with and without seizures is described in table 2. Of the 59 neonates with moderate HIE, 27 (46%) had seizures while 17 of the 24 (71%) neonates with severe HIE had seizures. There were also relatively high number of neonates with metabolic/genetic disorders (16/20, 80%) and stroke (10/18, 56%) who had seizures. Six neonates had ongoing electrographic seizures at the start of EEG monitoring, and in this group the EEG and study enrolment tended to be late—at a median age of 19.8 hours (IQR 6.7–55.8). Of those, three had a diagnosis of stroke, two HIE (one with stroke and moderate HIE) and one had a metabolic/genetic disorder.

**Table 2:** Aetiology by seizure status group, n=214

	n	Seizures on EEG	No seizures on EEG	
			Did not receive AED before EEG start (n=115)	Received AED before EEG start (n=24)
		n (%)	n (%)	n (%)
HIE grade				
Mild	50	0 (0.0)	48 (41.7)	2 (8.3)
Moderate	59	27 (36.0)	23 (20.0)	9 (37.5)
Severe	24	17 (22.7)	3 (2.6)	4 (16.7)
Metabolic/genetic disorder	20	16 (21.3)	4 (3.5)	0 (0.0)
Stroke	18	10 (13.3)	5 (4.3)	3 (12.5)
Suspected seizures—unconfirmed	12	0 (0.0)	9 (7.8)	3 (12.5)
Perinatal asphyxia without clinical encephalopathy	7	0 (0.0)	7 (6.1)	0 (0.0)
Sepsis/meningitis	6	2 (2.7)	3 (2.6)	1 (4.2)
Intracranial haemorrhage	5	0 (0.0)	4 (3.5)	1 (4.2)
Other	13	3 (4.0)	9 (7.8)	1 (4.2)

The characteristics of the seizures according to the underlying diagnosis are described in table 3. The median period between the start of the first recorded seizure to the end of the last recorded seizure ‘seizure period’ was 15.9 hours (IQR 4.4–42.1). A statistically significant difference was not found across the diagnostic groups for seizure period, although seizures tended to persist longer in neonates with severe HIE (table 3).

**Table 3:** Seizure characteristics by aetiology, n=75

	All seizure babies (n=75)	Moderate HIE (n=27)	Severe HIE (n=17)	Metabolic/genetic disorder (n=16)	Stroke (n=10)	Other* (n=5)	P values†
	Median (IQR)	Median (IQR)	Median (IQR)	Median (IQR)	Median (IQR)	Median (IQR)	
Seizure period (hours)‡	15.9 (4.4 to 42.1)	8.7 (2.0 to 21.2)	33.5 (11.9 to 58.0)	25.0 (1.1 to 77.6)	22.1 (6.6 to 55.1)	14.1 (4.0 to 65.6)	0.068
Age (hours after birth) at first recorded seizure	19.4 (12.8 to 48.8)	16.5 (8.7 to 35.5)	15.2 (11.0 to 20.7)	56.6 (42.7 to 99.3)	25.4 (14.2 to 44.9)	31.8 (18.1 to 74.4)	0.001§
Total seizure burden (min)	68.5 (27.5 to 166.8)	48.5 (23.2 to 79.6)	112.8 (32.7 to 223.6)	39.9 (14.6 to 210.6)	149.6 (54.2 to 289.6)	117.3 (2.9 to 283.6)	0.060
Number of seizures	24.0 (9.0 to 51.0)	10.0 (4.0 to 32.0)	31.0 (21.0 to 57.5)	30.5 (7.0 to 56.8)	50.0 (15.0 to 166.5)	12.0 (2.0 to 210.0)	0.005¶
Median seizure duration (s)	109.0 (65.0 to 225.5)	143.0 (96.0 to 696.0)	109.0 (66.5 to 205.8)	93.8 (44.1 to 162.6)	104.0 (63.1 to 158.0)	70.0 (42.0 to 243.0)	0.069
Maximum hourly seizure burden (MSB) (min/hour)	21.3 (11.3 to 32.3)	18.0 (14.4 to 28.3)	24.7 (14.2 to 40.1)	13.4 (7.9 to 24.2)	30.3 (18.3 to 42.9)	20.7 (1.3 to 30.2)	0.063
Hour after birth in which MSB occurred	31.0 (17.0 to 61.0)	17.0 (12.0 to 43.0)	27.0 (18.5 to 49.0)	57.0 (43.8 to 145.5)	29.5 (20.8 to 65.0)	56.0 (29.5 to 102.0)	0.002**
Age (hours after birth) at last recorded seizure	55.2 (28.5 to 86.1)	34.4 (16.6 to 59.9)	54.3 (28.1 to 80.3)	71.9 (47.9 to 304.9)	58.4 (31.5 to 122.0)	56.6 (30.2 to 126.5)	0.008††

*n=2 for seizures of unknown origin; n=2 for sepsis/meningitis; n=1 for postnatal cardiorespiratory arrest (and severe HIE).

†From Kruskall-Wallis test, with ‘other’ excluded from the analysis.

‡Time from start of first EEG seizure to end of last EEG seizure.

§Pairwise comparisons revealed significant differences between the metabolic/genetic disorder group and both the moderate HIE (adjusted p=0.002) and the severe HIE (adjusted p=0.002) groups.

¶Pairwise comparisons revealed significant differences between the moderate HIE group and both the severe HIE (adjusted p=0.034) and the stroke (adjusted p=0.019) groups.

**Pairwise comparisons revealed significant differences between the metabolic/genetic disorder group and the moderate HIE group (adjusted p=0.001).

††Pairwise comparisons revealed significant differences between the metabolic/genetic disorder group and the moderate HIE group (adjusted p=0.005).

EEG, electroencephalography; HIE, hypoxic-ischaemic encephalopathy.

The median age at the time of the first recorded EEG seizure was 19.4 hours (IQR 12.8–48.8). Age at first recorded seizure differed by aetiology (p=0.001). Neonates with a metabolic/genetic disorder were significantly older at first recorded seizure than both neonates with moderate (adjusted p=0.002) and severe HIE (adjusted p=0.002).

The median length of time from commencement of EEG to identification of first seizure was 6.0 hours (IQR 1.4–14.6). The time at which the first seizure was seen did not differ significantly (p=0.364) between the group who had been exposed to AEDs prior to the commencement of monitoring (median (IQR): 5.4 (1.9–28.9) hours, n=23) and those who had not (median (IQR): 6.0 (1.2–12.5) hours, n=52).

TSB ranged from 29 s to 15.18 hours with a median (IQR) of 68.5 (27.5–166.8) min. TSB was highest for neonates with stroke or severe HIE, but a statistically significant difference was not found between aetiologies (p=0.060). The number of seizures identified per neonate ranged from 1 to 551 with a median of 24 (IQR 9–51). Number of seizures differed by aetiology (p=0.005). Neonates with moderate HIE had significantly fewer recorded seizures than both neonates with severe HIE (adjusted p=0.034) and neonates with stroke (adjusted p=0.019). The median seizure duration was 109 s (IQR 65–225.5). Median seizure duration was not significantly different between different aetiologies (p=0.069).

MSB occurred at a median of 31 hours (IQR 17–61) after birth and was significantly different between aetiologies (p=0.002). MSB occurred significantly later for neonates with a metabolic/genetic disorder compared with neonates with moderate HIE (adjusted p=0.001). MSB ranged from 29 s/hour to 60 min/hour with a median (IQR) of 21.3 min/hour (11.3–32.3). Although MSB was highest for neonates with stroke or severe HIE, a significant difference was not found for aetiology (p=0.063). Twenty-one neonates with seizures (28%) had SE (MSB≥30 min/hour).^13^ The aetiology of neonates with SE was as follows: severe HIE (n=7), moderate HIE (n=6), stroke (n=5, two with concomitant moderate HIE), metabolic/genetic disorder (n=2) and sepsis/meningitis (n=1). The last electrographic seizure was recorded at a median age of 55.2 hours (IQR 28.5–86.1) and was significantly different between final diagnosis groups (p=0.008). Neonates with a metabolic/genetic disorder were significantly older at last recorded seizure than neonates with moderate HIE (adjusted p=0.005).

### Seizure treatment

Over half (112/214, 52%) of the neonates were given at least one AED, administered at a median of 12.2 hours of age (IQR 5.5–37.5). Almost all (104/112, 93%) received phenobarbitone as their first-line treatment (dosage: 20 mg/kg for n=93 and 10 mg/kg for n=11), with the remaining eight receiving midazolam (n=4), lidocaine (n=2), levetiracetam (n=1) and paraldehyde (n=1). Forty-seven of the 112 neonates (42%) received their first AED before EEG monitoring commenced (median (IQR): 5 hours (2.7–13.5)). Twenty-one of these 47 neonates (45%) were treated before transfer to the monitoring centres and hence were treated on clinical suspicion of seizure. Two of these 47 neonates were treated on the basis of seizure recognition on aEEG (applied before full cEEG available). Neonates with EEG confirmed seizures were more likely to have received an AED before EEG monitoring commenced (23/75 (31%) vs 24/139 (17%), p=0.024). Of the 65 neonates who received their first AED after EEG monitoring commenced, the median time from EEG start to first AED was 5.0 hours (IQR 2.1–16.6).

#### Neonates without electrographic seizures

In total, 139 neonates did not have any recorded electrographic seizures. Of these, almost one-third (44/139, 32%) were administered an AED (table 4). The clinical diagnoses of these neonates were: moderate HIE (n=21), severe HIE (n=6), stroke (n=5), intracranial haemorrhage (n=4), mild HIE (n=3), suspected seizures—unconfirmed (n=3), sepsis/meningitis (n=1) and postnatal cardiorespiratory arrest/mild HIE (n=1). The median age at which they received their first AED was 10.9 hours (IQR 4.1–38.3 hours). The majority (33/44, 75%) received one type of AED, while nine neonates (20%) received two AEDs and two neonates (5%) received three AEDs. Over half (24/44, 55%) of these neonates received their first AED before EEG monitoring commenced. Twenty-seven neonates who did not have any electrographic seizures received an AED after EEG monitoring commenced, including seven neonates who had also received an AED before the start of EEG monitoring. A total of 42 loading doses were given to the 27 neonates after EEG commencement, and the indications for AED use were: clinical seizure (n=29), suspected seizures (n=6), EEG seizure (n=2), aEEG seizure (n=1) and unknown (n=4). We do not know if the EEG was misinterpreted in these cases or simply not used. However, only one EEG had a rhythmic artefact present due to prominent respiration that may have been misinterpreted as a seizure.

**Table 4:** Neonates without electrographic seizures on the EEG, n=139

	Never received AED (n=95)	Received AED at any time (n=44)	Received AED before start of EEG monitoring (n=24)	Received AED after start of EEG monitoring (n=27)*
	n (%)	n (%)	n (%)	n (%)
Final diagnosis				
HIE grade				
Mild	47 (49.5)	3 (6.8)	2 (8.3)	1 (3.7)
Moderate	11 (11.6)	21 (47.7)	9 (37.5)	15 (55.6)
Severe	1 (1.1)	6 (13.6)	4 (16.7)	3 (11.1)
Metabolic/genetic disorder	4 (4.2)	0 (0.0)		
Stroke	3 (3.2)	5 (11.4)	3 (12.5)	4 (14.8)
Suspected seizures—unconfirmed	9 (9.5)	3 (6.8)	3 (12.5)	1 (3.7)
Sepsis/meningitis	3 (3.2)	1 (2.3)	1 (4.2)	0 (0.0)
Intracranial haemorrhage	1 (1.1)	4 (9.1)	1 (4.2)	3 (11.1)
Perinatal asphyxia without clinical encephalopathy	7 (7.4)	0 (0.0)		
Other	9 (9.5)	1 (2.3)	1 (4.2)	0 (0.0)
Type of AED†				
Phenobarbital		42 (95.5)	22 (91.7)	25 (92.6)
Midazolam		7 (15.9)	3 (12.5)	4 (14.8)
Phenytoin		3 (6.8)	3 (12.5)	0 (0.0)
Liddocaine		2 (4.5)	0 (0.0)	2 (7.4)
Clonazepam		2 (4.5)	2 (8.3)	0 (0.0)
Bumetanide		1 (2.3)	0 (0.0)	1 (3.7)
Number of types of AEDs administered†				
1		33 (75.0)	19 (79.2)	23 (85.2)
2		9 (20.5)	4 (16.7)	3 (11.1)
3		2 (4.5)	1 (4.2)	1 (3.7)

*Includes seven neonates who also received AED before start of EEG monitoring.

†For neonates who were administered an AED.

AED, antiepileptic drug; EEG, electroencephalography; HIE, hypoxic-ischaemic encephalopathy.

#### Neonates with electrographic seizures

The majority of neonates (61/75, 81%) with electrographic seizures were administered an AED during EEG monitoring (table 5). Seven neonates never received an AED at any time, while an additional seven received an AED before commencement of the EEG only and subsequent seizures were not treated. TSB, MSB and number of seizures were significantly higher in the seizure neonates who received an AED during EEG monitoring (n=61) compared with seizure neonates who did not (n=14). The seizure period was also significantly longer in the group that received an AED (table 6). The TSB in the group with seizures which were not treated during the recording (n=14) ranged from 29 s to 4 hours and 47 min with a median (IQR) of 33.2 (1.2–104.4) min. A review of all the seizures in these 14 babies revealed that the vast majority of seizures were low amplitude on the EEG and showed little or no change on the aEEG. It follows, that if the local practice was to use aEEG to screen for areas of EEG interest to review, these seizures would have been missed.

**Table 5:** AEDs administered to neonates with electrographic seizures on the EEG, n=75

	Never received AED (n=7)	Received AED at any time (n=68)	Received AED before start of EEG monitoring (n=23)	Received AED after start of EEG monitoring (n=61)*
	n (%)	n (%)	n (%)	n (%)
Final diagnosis				
HIE grade				
Moderate	4 (57.1)	23 (33.8)	9 (39.1)	18 (29.5)
Severe	1 (14.3)	16 (23.5)	4 (17.4)	16 (26.2)
Metabolic/genetic disorder	1 (14.3)	15 (22.1)	5 (21.7)	14 (23.0)
Stroke	0 (0.0)	10 (14.7)	2 (8.7)	10 (16.4)
Sepsis/meningitis	0 (0.0)	2 (2.9)	2 (8.7)	2 (3.3)
Other	1 (14.3)	2 (2.9)	1 (4.3)	1 (1.6)
Type of AED†				
Phenobarbital		65 (95.6)	23 (100.0)	56 (91.8)
Midazolam		19 (27.9)	1 (4.3)	18 (29.5)
Phenytoin		25 (36.8)	1 (4.3)	24 (39.3)
Lidocaine		6 (8.8)	1 (4.3)	5 (8.2)
Clonazepam		5 (7.4)	1 (4.3)	4 (6.6)
Keppra/levetiracetam		3 (4.4)	0 (0.0)	3 (4.9)
Paraldehyde		4 (5.9)	0 (0.0)	4 (6.6)
Bumetanide		3 (4.4)	0 (0.0)	3 (4.9)
Pyridoxal 5 phosphate		2 (2.9)	0 (0.0)	2 (3.3)
Pyridoxin		3 (4.4)	0 (0.0)	3 (4.9)
Vigabatrin		2 (2.9)	0 (0.0)	2 (3.3)
Number of types of AEDs administered†				
1		23 (33.8)	19 (82.6)	20 (32.8)
2		28 (41.2)	4 (17.4)	25 (41.0)
3		12 (17.6)	0 (0.0)	12 (19.7)
≥4		5 (7.4)	0 (0.0)	4 (6.6)

* includes 16 neonates who also received an AED before the start of EEG monitoring.

†For neonates who were administered an AED.

AED, antiepileptic drug; EEG, electroencephalography; HIE, hypoxic-ischaemic encephalopathy.

**Table 6:** Seizure characteristics split by AED group, n=75

	Received AED during EEG monitoring (n=61)	Did not receive AED during EEG monitoring (n=14)	P values*
	Median (IQR)	Median (IQR)	
Seizure period (hours)†	22.1 (6.7 to 50.0)	8.0 (0.03 to 15.8)	0.002
Total seizure burden (min)	75.1 (31.2 to 176.8)	33.2 (1.2 to 104.4)	0.030
Number of seizures	28.0 (12.0 to 52.0)	4.5 (1.8 to 20.8)	0.003
Median seizure duration (s)	108.0 (64.5 to 223.0)	113.0 (66.0 to 293.5)	0.849
Maximum hourly seizure burden (min/hour)	23.0 (14.1 to 32.4)	12.1 (1.2 to 29.2)	0.037

*From Mann-Whitney U test.

†Time from start of first EEG seizure to end of last EEG seizure.

AED, antiepileptic drug; EEG, electroencephalography.

The median age at first AED was 13.0 hours (IQR 7.6–37.2). The majority (51/68, 75%) received either one or two types of AED. Almost all neonates were administered phenobarbitone as their first-line treatment (65/68, 96%; dosage: 20 mg/kg for n=55 and 10 mg/kg for n=10).

One-third (23/68, 34%) of neonates with electrographic seizures who were administered an AED received it before EEG monitoring commenced. Sixty-one of the 68 neonates with electrographic seizures received an AED after EEG monitoring commenced, including 16 neonates who also received AED before the start of the EEG monitoring. The number of different AED medications received after EEG commencement ranged from 1 to 6 with a median of 2 (IQR 1–3). Of the 45 seizure neonates who received their first AED after EEG monitoring commenced, 8 received it before the first recorded seizure and the remaining 37 neonates received it at a median time of 2.0 hours (IQR 0.8–4.2) after their first recorded seizure.

**Figure 2:**
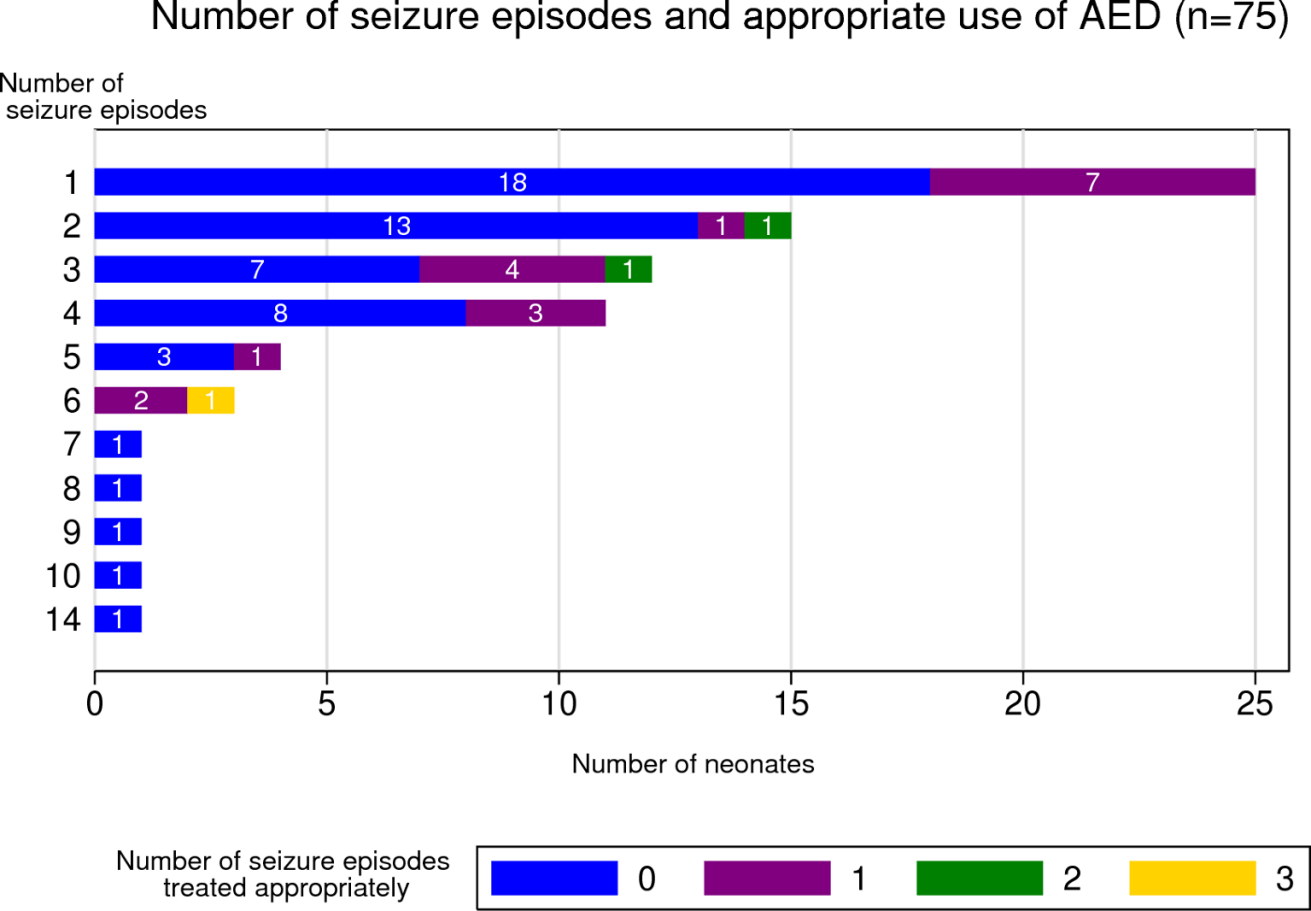
Number of seizure episodes and use of antiepileptic drugs (AEDs). The first bar illustrates that 25 neonates experienced one seizure episode and of those, 7 neonates had the seizure episode treated appropriately. The third bar illustrates that 12 neonates experienced three seizure episodes and of those, 1 neonate had two episodes treated appropriately, 4 neonates had one episode treated appropriately and 7 had none of their three episodes treated appropriately.

Across the 75 neonates with seizures, a total of 221 seizure episodes occurred and 11% (24/221) were treated with an AED within 60 min of the start of the seizure episode. The number of seizure episodes ranged from 1 to 14 with a median of 2 (IQR 1–4). Fifty-four (72%) neonates with seizures (median number of seizure episodes 2 (IQR 1–4), range 1–14) did not receive an AED at the appropriate time for any seizure episode, seven of whom did not receive an AED at any time. Seven (out of 25) neonates with a single seizure episode had that episode treated appropriately. Fourteen neonates with more than one seizure episode had at least one episode treated appropriately, with the percentage of seizure episodes appropriately treated ranging from 17% to 67% with a median (IQR) of 33% (24%–50%), figure 2. When we examined each entire seizure episode, 39% (86/221) were treated with an AED at some point during the episode or within 60 min of the end of the seizure episode.

## Discussion

The findings of this study reflect current practice in European neonatal units that have access to cEEG monitoring. In neonates in whom EEG monitoring was clinically required, electrographic seizures were present in over a third. Accurate quantification of the number and duration of electrographic seizures by expert reviewers revealed the high seizure burden in many cases, even in spite of treatment. Seizures were generally seen after about 6 hours of recording when present. In neonates with HIE, seizures reached a peak between 17 (moderate) and 27 (severe) hours after birth. Overall, seizures tended to be present for longer periods (seizure period) in those with severe HIE. These findings confirm the pattern of seizure evolution in HIE, which we and others have previously described.^14^ This information is useful clinically; if the evolution is not as expected then an alternative diagnosis should actively be considered, and seizures which persist beyond 96 hours should be a trigger for additional investigations.

In spite of treatment with AEDs, the seizure burden remained high with 28% meeting the criteria for neonatal SE, namely electrographic seizures continuing for more than half of any hour. Neonates with stroke and severe HIE tended to have the largest seizure burden. The resistant nature of neonatal seizures is shown by the number of AEDs which were given to neonates with seizures; 60% of neonates were given more than one type of AED and 23% received three or more. A few neonates may have responded to the AED administered before monitoring started, given that 24 were given an AED before the commencement of cEEG monitoring and then never had an electrographic seizure. In 2/24, aEEG monitoring started prior to cEEG monitoring. Ictal discharges may have been recognised on the aEEG and treated prior to cEEG monitoring.

The collaborators were all experienced users of neonatal cEEG and aEEG before the study commenced. Despite this, the use of AEDs was not always timely or appropriate, reflecting the difficulty in interpretation of EEG round the clock in NICUs. Fourteen neonates who had seizures on their cEEG were never treated during monitoring, and 27 neonates whose cEEG did not ever show seizures were given AEDs while the EEG was running. These findings reflect the real-world application of cEEG and are likely to be similar in other units. Clinicians are faced with having to review many hours of cEEG at a time rapidly, and they tend to use the aEEG as a guide to where to interrogate the cEEG trace. aEEG is a useful tool which can be used to guide treatment,^10^ but aEEG can be poor at detecting short seizures, low voltage seizures and seizures which do not generalise.^15 16^ In addition, although all centres were experienced in using EEG, each had their own protocol for reviewing the aEEG. There are no definitive guidelines about how regularly cEEG or aEEG should be reviewed in neonates at risk of seizures.

The most common artefacts seen in the EEG are those due to respiration, ECG, sweat and patting, and these can be difficult for clinicians to identify.^17^ Although experts generally agree regarding the interpretation of a formal EEG, some seizure patterns are very difficult even for experts to recognise.^18^

Despite the fact that real-time cEEG monitoring was ongoing, when AEDs were given, they were not often given within 1 hour of an electrographic seizure occurring on the cEEG. Of the 221 seizure episodes which were captured, on only 24 (11%) of the occasions was an AED given within a 1 hour of seizure onset, suggesting that in clinical practice seizures are not recognised at the time they occur even when cEEG monitoring is in place. A further reason may be the time taken to prescribe, obtain and administer AEDs. It is not known if treating seizures promptly, if an effective treatment can be found, has the potential to reduce the TSB but is clearly an area for future research. This is also an area where clinical support tools such as seizure detection algorithms may help.^2^ These algorithms may not be perfect but can alert clinical teams when suspicious EEG activity occurs so that the cEEG is reviewed as soon as possible.

This study has a number of limitations. First, even though seizures were annotated by a group of board-certified electroencephalographers with specific experience in neonatal EEG, each EEG was not annotated by all four. Therefore, we do not have data on inter-rater agreement. However, we and others have previously shown that agreement for neonatal EEG seizure identification between expert reviewers is very high.^18^ Second, review of cEEG at each site was ad hoc and dependent on the individual neonate. There are no protocols in place for review of cEEG at any site as neurophysiology services are not available 24/7. Clinicians request review when concerns are raised on EEG or clinically for signs of seizures.

Third, while protocols do exist for the type of first-line, second-line and third-line AEDs to be used for the treatment of neonatal seizures, the recommended timing of administration of the AED is never stipulated. This is largely due to the fact that this detailed information has never been available before, that is, detailed EEG seizure information. In the absence of any clear guidelines for the optimal timing of AED administration, we arbitrarily expected that seizures should be treated within 1 hour of onset in order to be considered as having been appropriately treated. We applied this definition consistently to each EEG from each infant.

## Conclusion

Retrospective review of cEEG recordings in six European NICUs revealed a high seizure burden in many neonates, irrespective of aetiology. Neonatal seizures generally begin early after birth in full-term neonates, persist for several days and remain relatively resistant to multiple AEDs. Despite the availability of both cEEG and aEEG in our centres, many seizures were not treated and other non-seizure events were treated with AEDs. Only 11% of all electrographic seizure episodes were treated within 1 hour of onset. This highlights the difficulties encountered in interpreting the neonatal EEG in real time in the NICU. There is an urgent need for automated decision support tools to help detect neonatal seizures so that they can be treated more accurately and promptly.
